# AOM/DSS Induced Colitis-Associated Colorectal Cancer in 14-Month-Old Female Balb/C and C57/Bl6 Mice—A Pilot Study

**DOI:** 10.3390/ijms23095278

**Published:** 2022-05-09

**Authors:** Martin Schepelmann, Nadja Kupper, Valeriya Gushchina, Ildiko Mesteri, Teresa Manhardt, Stefan Moritsch, Christian Müller, Karina Piatek, Martina Salzmann, Andrea Vlasaty, Robert Eferl, Enikö Kallay

**Affiliations:** 1 Institute for Pathophysiology and Allergy Research, Center of Pathophysiology, Infectiology & Immunology, Medical University of Vienna, 1090 Vienna, Austria; nadjakupper@googlemail.com (N.K.); valeriya.gushchina@meduniwien.ac.at (V.G.); teresa.manhardt@meduniwien.ac.at (T.M.); christian_muell_er@hotmail.com (C.M.); karina.piatek@meduniwien.ac.at (K.P.); martina.salzmann@meduniwien.ac.at (M.S.); andrea.vlasaty@meduniwien.ac.at (A.V.); enikoe.kallay@meduniwien.ac.at (E.K.); 2 Pathologie Überlingen, 88662 Überlingen, Germany; ildiko.mesteri@hotmail.com; 3 Center for Cancer Research, Medical University of Vienna, 1090 Vienna, Austria; stefan.moritsch@meduniwien.ac.at (S.M.); robert.eferl@meduniwien.ac.at (R.E.)

**Keywords:** aged mice, colitis associated colorectal cancer, AOM, DSS, mouse strain differences, pilot study

## Abstract

Colitis is a major risk factor for the development of colorectal cancer, leading to colitis-associated colorectal cancer (CAC). The most commonly used animal model to study CAC is the azoxymethane-dextran sulphate-sodium (AOM/DSS) model. The ideal experimental conditions of this model depend on several factors, including the used mouse strain. No data on feasibility and conditions for older mice, e.g., for aging studies, have yet been reported. Thus, we conducted a descriptive, observational pilot study where CAC was induced in 14-month-old female Balb/C and C57/Bl6 mice using 12.5 mg/kg AOM i.p. and three different concentrations of DSS (1, 2, and 3%) in drinking water (ad. lib.). The mice were monitored regularly during the three-month experimental phase. After euthanasia, the colons of the mice were evaluated macroscopically and microscopically. Both the mouse strains showed a DSS-concentration-dependent induction of CAC. Carcinomas were only observed at 3% DSS. The DSS dose was found to be significantly correlated with the histology score and % Ki67 positive cells only in C57/Bl6 mice but not in Balb/C mice, which showed a variable response to the CAC induction. No differences in colon length, weight, or mucin content were observed. Optimal conditions for CAC induction in these aged animals are thus considered to be 3% DSS, as carcinomas did not develop when 2% DSS was used. On the other hand, Balb/C mice reacted severely to 3% DSS, indicating that 2.5% DSS may be the “sweet spot” for future experiments comparing CAC in aged Balb/C and C57/Bl6 mice. This model will allow investigation of the effect of aging on CAC development and therapy.

## 1. Introduction

The standard animal model for colitis-associated colorectal cancer (CAC) is the Azoxymethane (AOM)/Dextran-Sulphate-Sodium (DSS) mouse model, which is used to evaluate nutritional and pharmacological interventions for CAC [1,2]. The pathogenesis of this model resembles that of human colorectal tumors, as it recapitulates the adenoma-carcinoma sequence, but these tumors are rarely invasive [3,4]. The AOM/DSS model has been extensively used to study disease progression and nutritional or pharmacological interventions to treat or prevent CAC or to study risk factors associated with it [5,6]. However, these experiments are almost exclusively carried out in young animals, generally ranging from 5 to 12 weeks, which corresponds to around 10–25 years in humans [7].

Aging significantly increases the vulnerability to gastrointestinal disorders, and approximately 40% of geriatric patients report at least one GI complaint during a routine physical examination [8]. Colorectal cancer is predominately a disease of elderly patients [9], as the median age at diagnosis of colon cancer is 67 years [10]. This corresponds to around 17 months of age in mice [7].

Pharmacology, dosing of chemotherapeutic drugs, etc., are all different in the elderly than in young patients [9]. This is also true in aged animals, as metabolism markedly changes in aging mice [11]. Thus, most preclinical colorectal cancer studies are being performed on young mice but ultimately aim to simulate the disease in elderly patients. Ideally, these studies would be performed on animals corresponding to the same age as the patients at the median age of diagnosis. However, studies on aged mice are already challenging for a variety of reasons, including complex planning, costly housing arrangements, and the shrinking of the aging colony due to natural loss. The AOM/DSS experimental model of CAC takes around 3 months from the first injection to euthanasia and organ harvest and can induce a further reduction of colony size due to the animals’ reaching humane endpoints, further exacerbating these problems.

The aim of our study was, on the one hand, to test whether it is feasible to induce CAC in aged mice older than 14 months (corresponding to a 70–75-year-old human, thus better reflecting the human disease) and whether these old animals would survive the long treatment period. On the other hand, we wanted to assess whether low-level inflammation (1% DSS) would be enough to support tumor progression. Therefore, we performed a descriptive pilot study to assess the experimental conditions necessary to induce CAC in aged mice of the two most commonly used experimental mouse strains, Balb/C and C57/Bl6. Here, we show that aged animals of both mouse strains can indeed be used for AOM/DSS-induced CAC studies. The use of such old animals will allow the investigation of aging on the development and phenotype of CAC and the investigation of CAC progression and intervention in a model closer to the modeled human population segment.

## 2. Results

**Figure 1:**
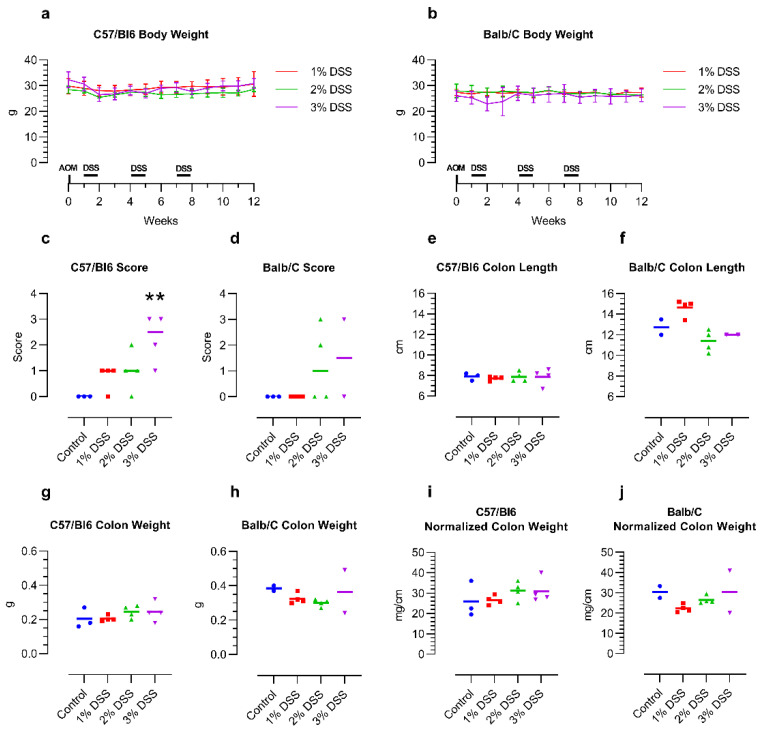
Effect of DSS treatment on body weight (**a**,**b**), histology score (**c**,**d**) and colon macroscopic parameters (**e**–**j**) of 14-month-old female C57/Bl6 and Balb/C mice with CAC induced by 3 different concentrations of DSS. Control = untreated control mice of the same age. *n* = 2–4. Histology score: 0—no abnormalities, 1—minimal changes such as aberrant crypt foci, 2—high-grade dysplasia, 3—intramucosal carcinoma. ** *p* < 0.01 Kruskal–Wallis with Dunn post hoc test vs. control for C57/Bl6. Statistical correlation analysis is provided in Figure 3.

The weight of the mice stayed relatively stable from before AOM injection until the end of the 12-week tumor induction period, independent of the DSS concentration or mouse strain. Treatment with 3% DSS led to intermittent weight loss, especially in the Balb/C mice (Figure 1a,b). 

After the first DSS treatment period, two Balb/C mice receiving 3% DSS had to be euthanized due to reaching the humane endpoint. For the remaining two mice in this group, the DSS dose was thus reduced to 2.5% for the second and third rounds of DSS treatment. In the control group (*n* = 3), receiving no AOM/DSS, one Balb/C mouse died from natural causes during the three months of the experimental group’s tumor induction. Of the C57/Bl6 mice, none of the mice reached a higher level of severity during the treatment.

The histology score of the tumors increased with the concentration of the DSS in the drinking water of the mice, though the C57/Bl6 mice appeared to react more strongly to the DSS than the Balb/C mice. While 1% DSS did not induce any morphological changes in the Balb/C mice, aberrant crypt foci were already visible in the C57/Bl6 mice. The higher concentrations of 2–3% DSS led to the development of up to high-grade dysplasia and even carcinomas in some animals in both mouse strains. Interestingly, one of the remaining Balb/C mice treated with 3% and then two times with 2.5% DSS did not develop any abnormalities at all, despite the apparent stronger effect of the treatment on the mice themselves (Figure 1c,d; representative images: Figure S1). Colon length, weight, and normalized colon weight (weight per length) did not show any obvious changes over untreated control mice, which indicates that the inflammation induced by the DSS had remitted in the 4 weeks after the final DSS treatment (Figure 1e–j). 

**Figure 2:**
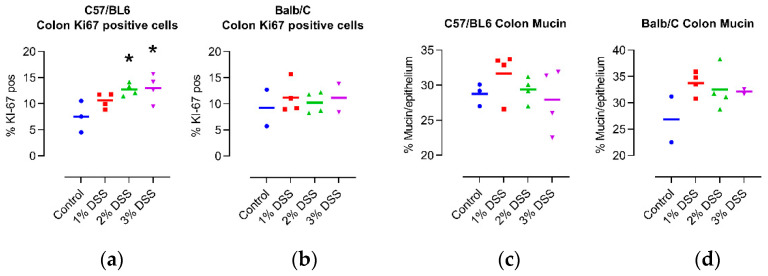
Effect of DSS treatment on percentage of Ki67 positive cells (**a**,**b**), and mucin content (**c**,**d**) in colons of 14-month-old female C57/Bl6 and Balb/C mice with CAC induced by 3 different concentrations of DSS. Control = untreated control mice of the same age. N = 2–4. * *p* < 0.05, ANOVA with Dunnett post hoc test vs. Control for C57/Bl6. Statistical correlation analysis is provided in Figure 3.

In the colons of C57/Bl6 mice, the percentage of KI67 positive was strongly dependent on the amount of DSS in the drinking water, while only a minor difference was seen in the colons of Balb/C mice (Figure 2a,b; representative micrographs: Figure S2), fitting with the results of the histology score. Based on the quantification of mucin in the colons of the mice, we found that the intensity of the DSS treatment had no significant effect on protective mucus production (Figure 2c,d; representative images: Figure S3), again pointing toward the regeneration of the mucosa from the inflammatory state, in which mucin is lost, in the 4-week period following the last DSS cycle. 

**Figure 3:**
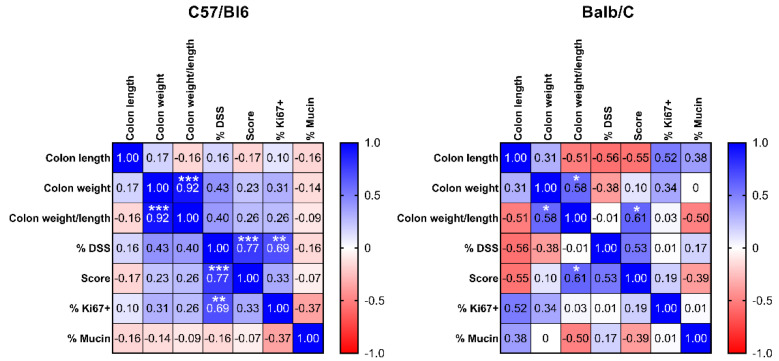
Spearman correlation matrix displaying Spearman correlation coefficient *r* (*r* = 1: 100% positive correlation, *r* = 0: no correlation, *r* = −1: 100% negative correlation) for all pairs of variables measured in C57/Bl6 (*n* = 15) and Balb/C (*n* = 12) mice (including non-treated control mice). Blue = positive correlation, white = no correlation, red = negative correlation. * *p* < 0.05, ** *p* < 0.01, *** *p* < 0.001 significance for correlation.

Correlation analysis of the investigated parameters revealed similar directions of correlation between parameter pairs for both mouse strains, although only a few of them reached statistical significance (Figure 3). In C57/Bl6 mice, we observed a highly significant positive correlation between the concentrations of DSS in the drinking water and both histology score and the percentage of Ki67 positive cells, indicating that the amount of DSS administered to these animals significantly affected both colon remodeling/development of malignancy and cell proliferation. In the Balb/C mice, we found no statistically significant correlation between the concentration of DSS and the histology score (*p* = 0.109) as a primary readout, although the direction was the same as in the C57/BL6 mice. This might be because two of the mice in the 3% DSS group died after the first treatment, and only two survived. Interestingly, in the Balb/C mice, normalized colon weight showed a significant positive correlation with the histology score, indicating a higher weight per cm with increasing dysplasia/tumor burden. This is indicative of a higher tissue density, probably due to the malignant tissue transformation. Normalized colon weight was significantly correlated with colon weight, as expected since the parameters are derived from each other. 

## 3. Discussion

Data on the differences between young and aged mice are still limited, although studies in aging mice are highly relevant as their metabolism and physiology change over time, as does that of the humans they serve as models for. Knowledge of the impact of aging on the GI tract of naturally aged mice is still limited; only a few studies are available. A recent study [12] found that aging in C57Bl/6 male mice led to thinning of the colonic mucus layer, reduction of goblet cell number, and increased bacterial permeability. The diversity of the fecal microbiota was increased in the old mice, while B cell activity and host immune responsiveness were decreased [12]. Similar results were seen also in female mice [13]. 

Despite these age-dependent changes, there are very few intervention studies on old mice. One recent study investigated the effect of the duration of tumor development post-tumor induction with AOM, where the mice were in the longest cohort at around 9-months-old at the time of sacrifice [14], which still only corresponds to ~35 years of age in humans [7], while the median age at diagnosis for colon cancer is 67 years [10]. 

To investigate whether and how the above-mentioned age-related changes affect CAC development, it will be necessary to perform the experiments in aged mice and compare the results with those in young animals. To facilitate this, it was necessary to establish whether the AOM/DSS model of CAC was, in fact, feasible in aged mice. The major conclusion from our study is now that aged mice (older than 14 months) indeed respond to the AOM/DSS treatment with the formation of colorectal tumors, as do young mice. Therefore, there is no reason why they should not be used as a model for age-dependent diseases such as colorectal cancer. Similar to young mice, the condition of the intervention needs to be determined in a pilot study, as the sensitivity of the mice to AOM/DSS might be different compared with the young (3–5 months old) animals. Moreover, the sensitivity of the strain might change with age. In our study, 3% DSS-induced tumors in both strains but appeared to be too severe in the Balb/C mice. Thus, 2.5% DSS seems to be the “sweet spot” for comparing AOM/DSS-induced CAC interventions in aged Balb/C and C57/Bl6 mice. This is congruent with conditions used by us in the past [5].

One needs to keep in mind the biological variability incurred by these types of in vivo experiments and that it may be pertinent to consider non-responders when planning an experiment. A further important issue to keep in mind is to consider the probability of a higher mortality rate due to treatment-independent diseases that might occur due to the aging process.

In conclusion, in this pilot study, we have now demonstrated the possibility of using mice of advanced age for AOM/DSS-induced CAC studies in two of the most commonly used mouse strains. Future studies may use this information to perform intervention studies aimed at CAC in a mouse age group closer to the modeled population segment and to investigate the effect of aging on the development and phenotype of CAC.

## 4. Materials and Methods

### 4.1. Chemicals

All chemicals were bought from Merck (Darmstadt, Germany) unless otherwise stated.

### 4.2. Animals

All animal experiments were approved by the Ethics Committee of the Medical University of Vienna and the Austrian Federal Ministry of Education, Science and Research (GZ 2020-0.688.103) and carried out in accordance with the European Union Regulations on Care and Use of Laboratory Animals.

12-month-old female ex-breeder Balb/C and C57/Bl6 mice (Envigo, Venray, Netherlands) were group-housed on a 12/12 h light-dark cycle and allowed unrestricted access to food (LASQdiet^®^ Rod16-A, LASVendi GmbH, Soest, Germany) and water (standard Vienna tap water) for additional 2 months, bringing them to 14 months of age at the start of the experiment.

### 4.3. Induction of CAC

The experimental protocol was performed as described in [15] with few modifications. The injection solution for the AOM was prepared by spinning the brown glass vial containing 25 mg of pure AOM (Merck) under inert gas shortly in a centrifuge, breaking off the neck, and immediately adding 1 mL of sterile 0.9% physiological saline solution (Fresenius Kabi, Bad Homburg, Germany). The solution was then topped up to 10 mL with saline to achieve a final concentration of 2.5 mg AOM per ml of saline. 

**Figure 4:**
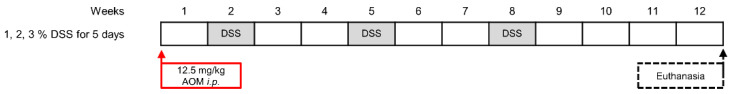
Schematic representation of the CAC induction protocol. In total, 12.5 mg/kg azoxymethane (AOM) were i.p. injected into the mice at the start of the protocol. In the second, fifth and eighth week, the drinking water of the mice was supplemented with 1, 2, or 3% (*w*/*v*) dextran sulphate sodium (DSS) for 5 consecutive days. 12 weeks after the AOM injection, the mice were euthanized.

All mice were weighed before the injection and the precise amount injected i.p. using 26G needles to achieve a dose of 12.5 mg/kg (5 µL injection per g body weight). After one week, the drinking water of the mice was supplemented with 1, 2, or 3% (*w*/*v*) of DSS (MP Biomedicals, Eschwege, Germany) for 5 days. After 2 weeks of recovery, this treatment was repeated two more times. For the Balb/C mice, the DSS concentration in the second and third cycles was reduced to 2.5% (*w*/*v*) as two of the mice were affected too strongly to carry on with the protocol and had to be euthanized. Four weeks after the third DSS treatment, the mice were euthanized, and their organs were removed for analysis; the mice were 17 months of age at this time. Mice were weighed weekly and monitored daily for their health and wellbeing. See Figure 4 for a scheme of the protocol. 

### 4.4. Colon Preparation

Colons were removed from cecum to anus, flushed with PBS, and their length and weight were measured. Colons were rolled in a Swiss roll and fixed in 4% neutral-buffered formaldehyde (Roti-Histofix, Carl Roth, Karlsruhe, Germany). The specimens were processed as per standard histology protocol, formalin-fixed, embedded in paraffin, and sliced into 4 µm sections.

### 4.5. Histology Scoring and Mucin Quantification

The sections were stained with hematoxylin and eosin and images were acquired using TissueFAXS Hard and Software (TissueGnostics GmbH, Vienna, Austria) using a 20× Objective (Neo-Fluar NA 0.5; Zeiss, Oberkochen, Germany). Scoring of the colons was carried out by an experienced pathologist under blinded conditions and was based on evaluation of the grade of malignant tissue transformation (score: 0—no abnormalities; 1—minimal changes such as aberrant crypt foci; 2—high-grade dysplasia; 3—intramucosal carcinoma). 

To evaluate mucosal barrier integrity, the amount of mucin per epithelium was stained and analyzed as described previously [16], with carcinoma areas excluded from the analysis.

### 4.6. Ki67 Immunostaining and Quantification

Paraffin-embedded colon sections were incubated for 25 min at 60 °C, deparaffinized, and rehydrated. After washing with PBS (pH 7.2), the sections were boiled in 0.05% citrate buffer for antigen retrieval, permeabilized with 0.2% Tween-20 in PBS for 20 min, and blocked with 5% goat serum in PBS for 30 min. The sections were then incubated with rat monoclonal anti-Ki-67 antibody-eFluor570 (eBioscience; 1:250 in 5% goat serum in PBS) antibody overnight at 4 °C. Nuclei were counterstained with DAPI (1:1000 in PBS) for 10 min at room temperature and samples were mounted using Fluoromount-G (Southern Biotech). Whole section immunofluorescence images were acquired with the automated TissueFAXS system (TissueGnostics GmbH). KI67 positive cells were analyzed using the TissueQUEST software (TissueGnostics GmbH) based on nuclear detection, counting KI67+ and KI67- cells as percentage of the total cell number in a section. 

### 4.7. Statistics

Data were analyzed and visualized using GraphPad Prism 9 (San Diego, CA, USA). Employed statistical tests are indicated at the respective figure legend.

## Data Availability

All data are reported in the manuscript.

## Supplementary Materials

The following supporting information can be downloaded at: https://www.mdpi.com/article/10.3390/ijms23095278/s1.
