# Supplementary material for: AOM/DSS Induced Colitis-Associated Colorectal Cancer in 14-Month-Old Female Balb/C and C57/Bl6 Mice—A Pilot Study
Source: Int J Mol Sci. 2022 May 9;23(9):5278. doi: 10.3390/ijms23095278 (PMC9104960; doi:10.3390/ijms23095278)
Supplement: Supplementary file 1 [file ijms-23-05278-s001.zip › ijms-1696447-supplementary.pdf]

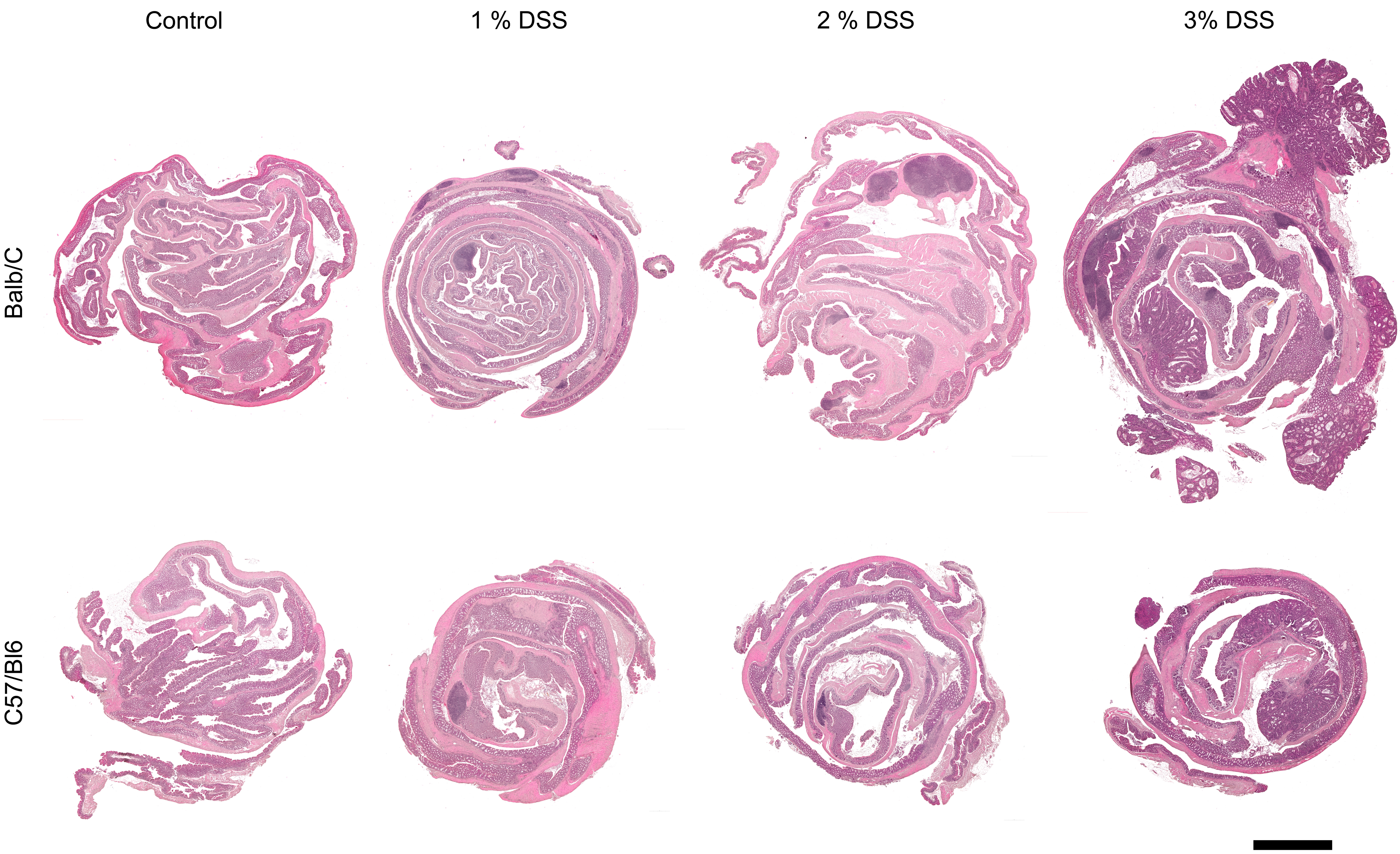

Figure S1: Representative H&E stainings of colon swiss rolls. Scale bar = 2 mm.

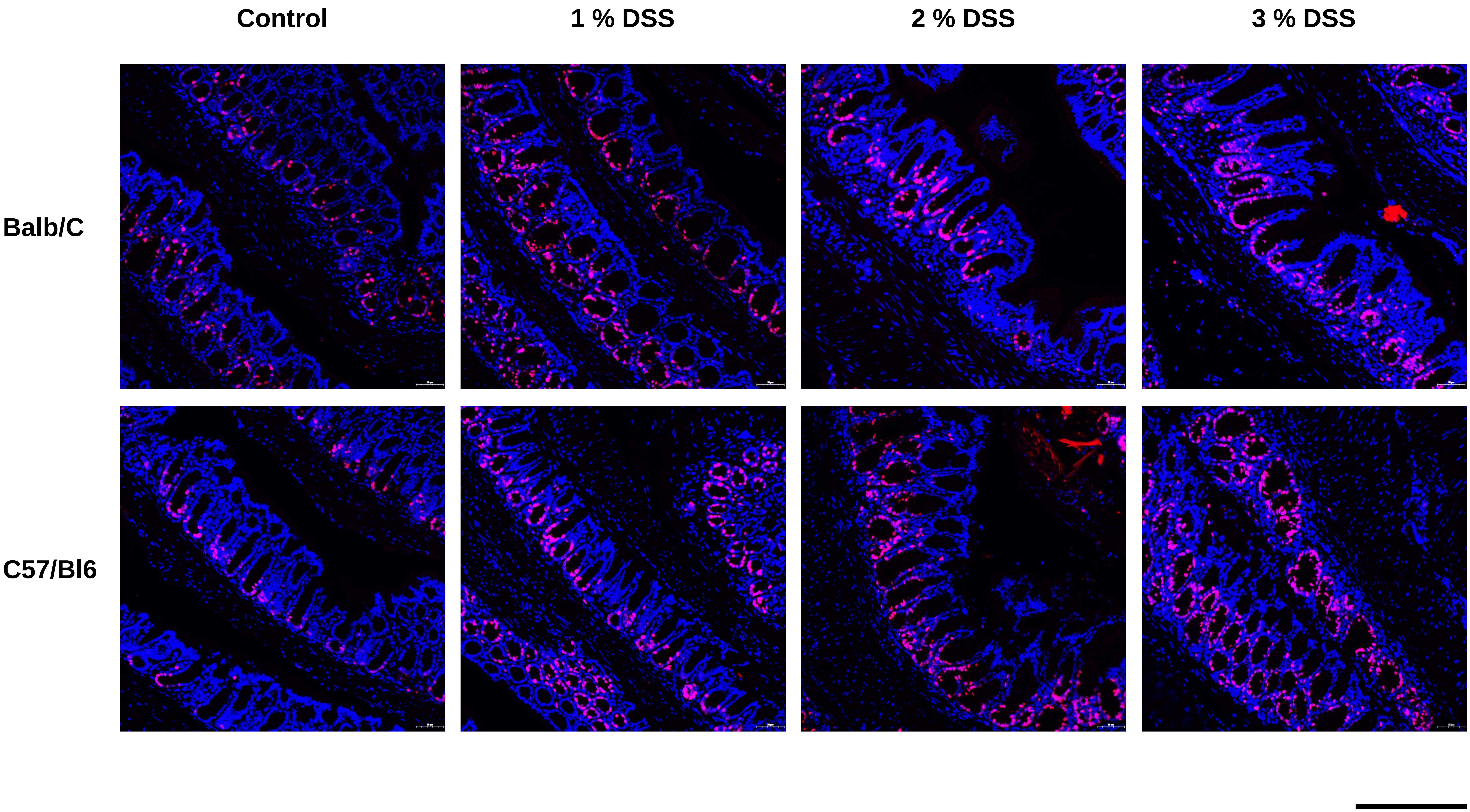

Figure S2: Representative immunofluorescence stainings for Ki67 (red), counterstained with DAP (nuclei, blue). Scale bar = 200 μm.

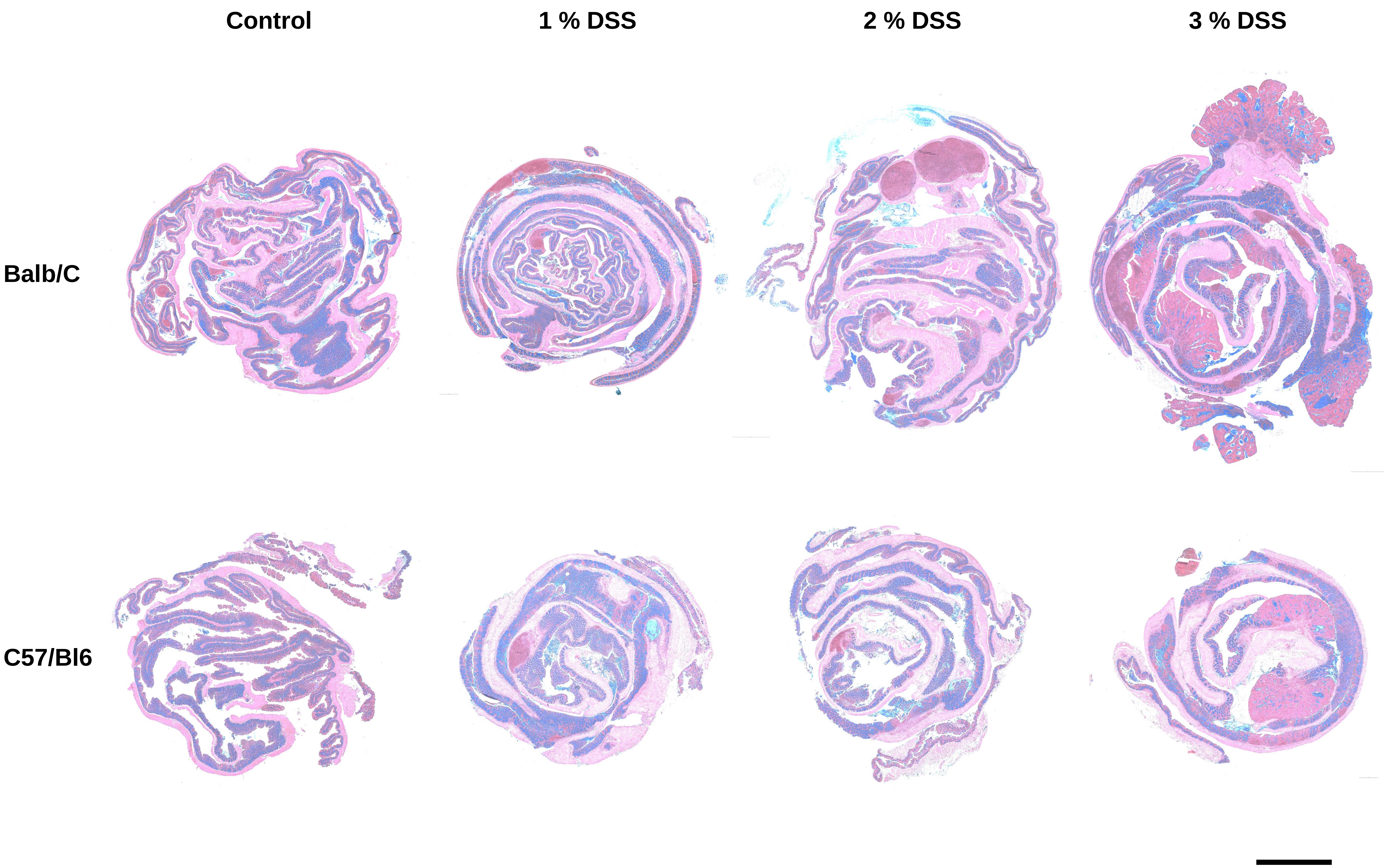

Figure S3: Representative mucin stainings (pink = tissue, blue = mucin). Scale bar = 2 mm.
